# Unique *Isospora*-associated histologic lesions in white-rumped shama (*Copsychus malabaricus*)

**DOI:** 10.1177/03009858221098425

**Published:** 2022-05-25

**Authors:** Talia S. Wong, Ilse H. Stalis, Carmel Witte, Steven V. Kubiski

**Affiliations:** 1 San Diego Zoo Wildlife Alliance, San Diego, CA; 2 University of California, Davis, Davis, CA

**Keywords:** avian, apicomplexan, *Atoxoplasma*, infectious disease, *Isospora*, passerine birds

## Abstract

Twenty-one white-rumped shamas (19 necropsied, 2 biopsied) (*Copsychus malabaricus*) housed at the San Diego Zoo between 1992 and 2020 were diagnosed with *Isospora* infection based on evaluation of histologic sections. Review of these cases revealed a consistent histologic lesion characterized by nodular aggregates of atypical epithelioid macrophages containing few intracytoplasmic protozoa, with or without lymphocytic infiltrates. Of the 19 necropsied cases, 16 (84%) had systemic lesions variably affecting the liver, spleen, gastrointestinal tract, lung, pancreas, connective tissues, or bone marrow, while all 21 diagnosed cases had skin involvement. The findings suggest that white-rumped shamas have a unique inflammatory response to isosporosis with a predilection for the skin. Skin may be a diagnostically sensitive sampling site for histologic diagnosis of *Isospora* in this species.

White-rumped shamas (*Copsychus malabaricus*) are small, Southeast Asian passerine birds common in aviaries. *Isospora* (formerly *Atoxoplasma*) is a genus in the family Eimeriidae comprised of ubiquitous apicomplexan protozoan parasites of passerine birds, closely related to *Eimeria*.^4,14^ The life cycle involves both sexual and asexual reproductive stages in the intestinal epithelium, producing unsporulated oocysts that are shed in the feces. In the environment, these sporulate to become infective to new hosts via ingestion. Some *Isospora* species have an additional asexual, replicative stage in circulating mononuclear cells. Many passerines have coevolved with their host-adapted *Isospora* species, and infections are typically subclinical and often diagnosed incidentally by fecal examination.^14^ If stressed or exposed to a high parasite load, fledglings may develop a severe and frequently fatal clinical syndrome characterized by lethargy, ruffled feathers, and diarrhea.^2,7,9,11,15^ This syndrome can be associated with severe gastrointestinal inflammation from sexual stages in the intestine, systemic inflammation from circulating replicative stages, or both. Several studies in black siskins (*Carduelis atrata*), canaries (*Serinus canarius*), Gouldian finches (*Chloebia gouldiae*), and song sparrows (*Melospiza melodia*) with this severe form of isosporosis describe a lymphocytic, lymphoproliferative, or lymphohistiocytic inflammatory response affecting multiple organs, particularly the small intestine, liver, spleen, kidney, and lung.^3,5,6,10,12^ In 2 of these studies, merozoites were shown by immunohistochemistry to parasitize lymphocytes specifically.^3,5^

In 1992, 3 female white-rumped shamas were imported from Hong Kong to the San Diego Zoo. On assessment, all 3 were diagnosed with subclinical *Isospora* by fecal examination and were released to the exhibit following treatment. Four fledglings hatched from one of these females died suddenly in 1996. At postmortem examination, all 4 were noted to have a unique, systemic histiocytic response and intracytoplasmic inclusions within mononuclear inflammatory cells that were suspicious for protozoal parasites. Skin, proventriculus, air sac, and aorta from 1 case (bird 1) were submitted to the Armed Forces Institute of Pathology (AFIP), and skin from a second case (bird 5) was submitted to the Davis branch of California Animal Health and Food Safety (CAHFS) for electron microscopy. Both laboratories identified the intrahistiocytic organisms as apicomplexan protozoa. Based on fecal examination, impression smears, histologic appearance, and electron microscopy interpretation, these organisms were determined to be consistent with *Isospora*.

To better characterize this atypical presentation of *Isospora* infection in white-rumped shamas, necropsy and biopsy records of white-rumped shamas housed at the San Diego Zoo between 1992 and 2020 were searched for cases that included the terms “Isospora” and “Atoxoplasma,” as well as white-rumped shamas with unexplained deaths, evidence of histiocytic inflammation, or skin lesions. Inclusion criteria for this study were defined as the presence of organisms consistent with *Isospora* identified on histology or cytology at the time of initial case submission and by secondary review. Twenty-one cases met the inclusion criteria, 19 of which were diagnosed histologically at necropsy and 2 of which were diagnosed by biopsy only. Polymerase chain reaction (PCR), which was not available at the time initial cases were identified, was performed on archived frozen tissues from 7 birds (cases 6, 12, 14, 16, 18, 19, and 20). Tissues tested included lung (n = 2), intestine (n = 6), liver (n = 2), heart (n = 1), or skin (n = 1) (see Supplemental Material 1). An additional 2 cases had *Isospora* diagnosed at necropsy but did not have further diagnostics or histologic sections available for review; these cases were excluded from the study summaries but are referenced in Supplemental Table S1.

Cases with *Isospora* infection included 6 females, 13 males, and 2 of undetermined sex. The age at diagnosis ranged from 20 days to 9.8 years (median age = 108.5 days), and 18 (86%) of 21 were diagnosed at less than 1-year-old by either necropsy or biopsy (median age in this subgroup = 88 days; range = 20–161 days). Splenomegaly was the most frequently reported gross finding at necropsy (5/19 necropsied cases, 26%). Hepatomegaly was uncommon (2/19 necropsied cases, 11%). No remarkable gross skin lesions were noted in deceased birds other than in cases coinfected with poxvirus. Skin biopsies were submitted due to nonspecific dermatologic lesions, including scabs and soft tissue swelling, and 2 biopsied cases had concurrent poxvirus infections (Supplemental Table S2). Other significant and common comorbidities in necropsied cases included poor body condition (6/19, 32%), trauma (7/19, 37%), candidiasis (3/19, 16%), aspergillosis (3/19, 16%), and avian poxvirus (3/19, 16%).

Histologically, *Isospora* infection was characterized by multiple nodular to coalescing aggregates of epithelioid macrophages within multiple tissues including liver, spleen, gastrointestinal tract, skin, lung, pancreas, connective tissues, and bone marrow (Figs. 1–6). Macrophages were prominent and characterized by abundant, pale, finely granular eosinophilic cytoplasm that frequently contained between 1 and 3 round-to-ovoid, poorly distinguishable merozoites (Fig. 7). Merozoites measured approximately 2–4 µm in diameter, with a basophilic nucleus and a faint, clear, perinuclear halo. Ziehl-Neelsen acid-fast stains were performed on 2 biopsies and 1 necropsy to rule out *Mycobacterium* sp. and were negative. Histiocytic aggregates were occasionally surrounded by lymphocytic cuffs, most prominently within the liver and spleen; however, lymphocytes were not consistently present. A grading scheme was developed to characterize the spectrum of *Isospora*-induced inflammation in the most consistently collected and most widely affected tissues, including liver, spleen, gastrointestinal tract, and skin (Supplemental Tables S3–S5). *Isospora*-associated lesions in the liver and spleen were graded into 4 categories based on the percentage of affected parenchyma, including severe (>60%), moderate (60%–30%), mild (10%–29%), and minimal (<10% or individual irregular histiocytes with intracytoplasmic protozoa). Sections of gastrointestinal tract were evaluated at 200× magnification and were graded into 3 categories from severe to mild based on numbers of macrophage aggregates within the field of view. Severe cases were defined as >5 aggregates per 200× field, moderate as 3–5 per 200× field, and mild as <3 per 200× field or individual, scattered histiocytes with intracytoplasmic protozoa. Skin lesions were qualitatively characterized as severe, moderate, mild, or minimal based on the degree to which nodules of affected macrophages coalesced and effaced the tissue structure. In all organs, the most severe lesion determined the final grade assignment.

**Figures 1–4.**
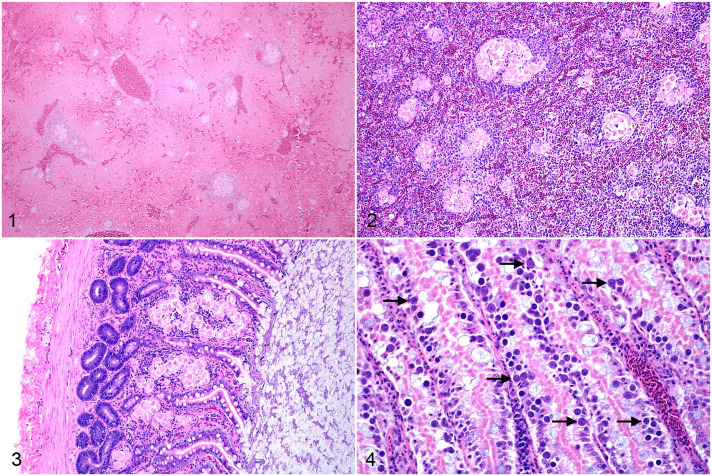
Systemic granulomatous inflammation due to *Isospora* infection, white-rumped shama. Hematoxylin and eosin. **Figure 1.** Liver, case 4. Disruption of the hepatic parenchyma by multifocal, discrete aggregates of *Isospora*-containing histiocytes. **Figure 2.** Spleen, case 5. Prominent, coalescing histiocytic aggregates in a severely affected spleen. **Figure 3.** Small intestine, case 5. Clusters of markedly distended histiocytes expand and distort the lamina propria. **Figure 4.** Isosporosis, small intestine, case 5. Innumerable sexual stages of *Isospora* spp. in the intestinal mucosa form round, roughly 20–30 µm diameter basophilic structures with a small central nucleus (presumed macrogamonts, arrows).

**Figure 5–7.**
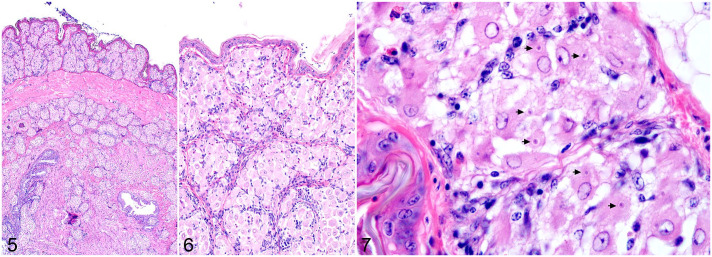
*Isospora* infection, skin, white-rumped shama, case 5. Hematoxylin and eosin. **Figures 5 and 6.** The dermis and subcutis contain extensive aggregates of epithelioid macrophages. **Figure 7.** The epithelioid macrophages contain numerous 2–4 µm diameter merozoites within the cytoplasm (arrows).

Gastrointestinal tract inflammation was typically granulomatous and composed of multiple aggregates of atypical, merozoite-filled macrophages within the serosa or lamina propria. Sexually replicative stages within enterocytes were only identified in 2 cases. Lack of available frozen tissues prevented molecular confirmation of these gametocytes as *Isospora* related to the systemic lesions. Lesions in either feathered or nonfeathered skin were identified in all 21 cases and were the only histologic evidence of *Isospora* infection in 5 cases. Inflammation within the skin was often extensive despite absent, minimal, or mild lesions within the remaining tissues. In 12 necropsy cases with available liver and lung impression smears, clear intramonocytic inclusions with nuclear indentation, consistent with *Isospora*, were identified in 10 cases. Poor cell preservation was a common complicating factor.

Outbreaks of isosporosis in young captive passerines are well-recognized and are suspected to coincide with periods of high stress, immunosuppression, or overcrowding that lead to high environmental parasite burdens, and thus high infectious doses when exposed upon fledging.^1,3,5–7,10,12,13^ There is limited and largely species-specific documentation of gross and histopathologic lesions associated with *Isospora* infection.^3,5,6,10,12^ In previously reported studies, there has been relative agreement on the typical gross and histologic findings, which include intestinal thickening, splenomegaly, and hepatomegaly or hepatopathy, and a primary lymphocytic to lymphohistiocytic response. The gastrointestinal tract has been the primary target organ in most of these previous cases, with intense and often transmural lymphocytic to lymphoproliferative infiltrates and smaller numbers of merozoite-containing lymphocytic cells in the spleen and liver.^3,5,6,10^ One report also found intraendothelial merozoites in green-winged saltators (*Saltator similis*).^8^ In our study, we observed that white-rumped shamas appear to have a unique, macrophage-dominant response associated with isosporosis. Lymphocytes were present in most but not all cases. Merozoites were reliably identified within macrophages and were not found in lymphocytes or any other cell. Inflammation targeted multiple organs with varying frequency, including the intestine, liver, and spleen. The skin was consistently affected in all examined cases, which has not been described in other passerines, and may prove a diagnostic site for both antemortem and postmortem detection of *Isospora* infection in this species. The shift in composition of *Isospora*-related inflammation in white-rumped shamas from lymphocytic to histiocytic may reflect a species-specific immune response to the parasite or may stem from antigens intrinsic to the infective *Isospora* species. Notably, only 2 birds in this study (both fledglings) had identifiable sexual stages of the organism in intestinal epithelium. Previous work suggests that these gametocytes likely represent the same organism as systemic merozoites.^14^ While the lack of gametocytes in most cases may be attributable to sampling error due to the segmental nature of intestinal *Isospora* infection, it is also possible that the species of *Isospora* affecting this population of white-rumped shamas represents a non-host-adapted strain, with exposure more commonly leading to systemic but nonpatent infection. Further studies examining the genetic and morphologic diversity of *Isospora*, host response, and cross-species infectivity are needed to better understand the pathogenesis of this parasite in both host-adapted and non-host-adapted infections.

## Supplemental Material

sj-pdf-1-vet-10.1177_03009858221098425 – Supplemental material for Unique Isospora-associated histologic lesions in white-rumped shama (Copsychus malabaricus) Click here for additional data file. Supplemental material, sj-pdf-1-vet-10.1177_03009858221098425 for Unique Isospora-associated histologic lesions in white-rumped shama (Copsychus malabaricus) by Talia S. Wong, Ilse H. Stalis, Carmel Witte and Steven V. Kubiski in Veterinary Pathology
