# Supplementary material for: Unique Isospora-associated histologic lesions in white-rumped shama (Copsychus malabaricus)
Source: Vet Pathol. 2022 May 25;59(5):869–72. doi: 10.1177/03009858221098425 (PMC9358608; doi:10.1177/03009858221098425)
Supplement: sj-pdf-1-vet-10.1177_03009858221098425 – Supplemental material for Unique Isospora-associated histologic lesions in white-rumped shama (Copsychus malabaricus) [file sj-pdf-1-vet-10.1177_03009858221098425.pdf]

Supplemental Material 1. PCR Methods.

DNA isolation: DNA was extracted from previously frozen feces and tissue samples. For the feces extraction, the Qiagen DNA Powersoil Kit (Valencia, CA) was utilized according to the manufacturer's protocol with the addition of a pretreatment step of 4 freeze-thaw cycles (3 min in liquid nitrogen followed by 3 minutes at 95°C). Tissue extraction was done with the Qiagen Dneasy Blood and Tissue Kit (Valencia, CA) following the manufacturer's tissue extraction protocol. Each sample DNA was eluted at 100 ul. The resultant DNA was stored short –term at -20°C and -80°C long-term.

Cytochrome c oxidase subunit I (COI) polymerase chain reaction assay (PCR): PCR assays were performed using the Eppendorf Mastercycler Pro S thermocycling system (Hauppauge, NY). The general procedure was taken from Ogedengbe et al. (2011) with forward primer Cocci_COI_For – 5'-GGTTCAGGTGTTGGTTGGAC -3' and reverse primer Cocci_COI_Rev - 5'- AATCCAATAACCGCACCAAG -3' for the cytochrome c oxidase subunit I (COI) target gene. Each 25 ul reaction contained the following reagents: 12.5 ul of 2X MyTaq HS Mix (Bioline, Memphis, TN), 400 nM of each primer (IDT, San Diego, CA), 2 ul of the tissue extracted DNA, and 4 ul of the fecal extracted DNA was added to each reaction. Each sample was run with a known positive control, negative extraction control, and PCR negative control. Controls were used to detect the success of the PCR as well as any contamination that might have occurred during the extraction or PCR set up. For the COI assay the following PCR parameters used were as follows 96°C for 5 minutes, [94°C for 20 seconds, 55°C for 30 seconds, 72°C for 90 seconds] x 40, 72°C for 10 minutes. The amplicons were run on a 0.9% agarose gel stained with 2.5% ethidium bromide. Bands of the expected size of ~780 bp were isolated and subsequently gel extracted using the EMD Millipore Ultrafree™-DA (Millipore, Billerica, MA).

Reference:
Ogedengbe, Joseph D., Robert H. Hanner, and John R. Barta. 2011. "DNA Barcoding Identifies Eimeria Species and Contributes to the Phylogenetics of Coccidian Parasites (Eimeriorina, Apicomplexa, Alveolata)." *International Journal for Parasitology* 41 (8): 843–50.

Supplemental Table S1. White-rumped shama necropsy cases diagnosed with *Isospora*; signalment and significant comorbidities.

Case Number	Sex	Age at Diagnosis (Days)	Euthanasia (Yes/No)	Body Condition	Spleno-megaly	Hepato-megaly	Significant Comorbidities
1	Female	730[a]	No	Fair	N	N	Trauma
2[b]	Female	1763	No	NR	N	N	Trauma, muscle necrosis
3	Male	20	No	Poor	N	N	Aspergillosus
4	Female	57	No	Good	Y	Y	Aspergillosis
5	Female	74	No	NR	Y	Y	Myocardial necrosis
6	Female	88	Yes	Poor	N	N	Aspergillosis
7	Male	48	No	Poor	N	N	Poor body condition
8	Male	101	No	Good	N	N	Trauma
9	Undetermined	161	No	Poor	N	N	None
10	Male	406	No	Good	Y	N	Trauma
11	Female	120	No	Poor	N	N	Poor body condition
12	Male	44	No	Good	N	N	Conjunctivitis
13	Male	149	No	Good	N	N	Trauma
14	Female	49	No	Good	Y	N	Candidiasis
15	Male	104	No	Fair	N	N	Myocarditis
16	Undetermined	38	No	Poor	Y	N	Poxvirus, candidiasis
17[b]	Female	508	No	Good	N	N	Trauma
18	Male	139	No	Fair	N	N	None
19	Male	146	No	Good	Y	N	Trauma
20	Male	3600	No	Good	N	N	Hypovitaminosis A
21	Male	113	No	Good	N	N	Anesthetic death

NR – not recorded. [a]Approximate age. [b]Slides unavailable for review.

Supplemental Table S2. White-rumped shama biopsy cases diagnosed with *Isospora*.

Biopsy Case	Age at Diagnosis (Days)	Tissue Submitted
B1[a]	113	Skin
B2	29	Skin
B3	1125	Skin

[a]Same individual as bird 21.

Supplemental Tables S3 – S5. Semi-quantitative grading scheme for histiocytic *Isospora*-associated lesions in white-rumped shamas.

S3. Liver, Spleen

Grade	Minimal	Mild	Moderate	Severe
% Tissue affected	< 10%	10% - 30%	30% - 60%	> 60%

S4. Gastrointestinal tract

Grade	Mild	Moderate	Severe
Histiocytic aggregates per 20x field (within lamina propria or serosa)	< 3	3 - 5	> 5

S5. Skin

Grade	Minimal	Mild	Moderate	Severe
Histologic Features	Rare histiocytic aggregates<br><br>No disruption of tissue architecture	Multifocal histiocytic aggregates without coalescing<br><br>Variable distortion of tissue architecture	Multifocal to coalescing histiocytic aggregates<br><br>Variable distortion of tissue architecture	Extensively coalescing histiocytic aggregates<br><br>Distortion of tissue architecture
